# Supplementary material for: Assessing geochemical and natural radioactivity impacts of Hamadat phosphatic mine through radiological indices
Source: PLoS One. 2023 Aug 3;18(8):e0287422. doi: 10.1371/journal.pone.0287422 (PMC10399901; doi:10.1371/journal.pone.0287422)
Supplement: Attachment — Submitted filename: PONE-D-22-34674 TII Report.pdf [file pone.0287422.s002.pdf]

Article

by Plos One

Submission date: 26-Dec-2022 07:37PM (UTC+0800)
Submission ID: 1986692126
File name: Manuscript_PONE_TII.docx (102.63K)
Word count: 4022
Character count: 21072

Implications of radiological indices of Hamadat phosphatic mine: Geochemical and Natural radioactivity aspects

Abstract: The present work manifests the geochemical characteristics, distribution of natural Radioactivity, and radiological risk assessment of the phosphorite deposits to assess their favorability in industrial applications such as preparing phosphoric acid and phosphatic fertilizers. The phosphorites are massive and collected from different beds within the Duwi Formation at the Hamadat mining area. They are grain-supported and composed of phosphatic pellets, bioclasts (bones), non-phosphatic minerals, and cement. Geochemically, phosphorites contain high concentrations of P_2O_5 (23.59- 28.36 wt.%) and CaO (40.85-44.35 wt.%), with low amounts of Al_2O_3, TiO_2, Fe_2O_3, Na_2O, K_2O, and MnO, suggesting the low contribution of the detrital material during their deposition. Moreover, they belong to uraniferous phosphorites due to a notably high U concentration (55-128 ppm). They are also enriched with Sr, Ba, Cr, V, and Zn and depleted in Th, Zr, and Rb, which strongly supports the low detrital input during the formation of the Hamadat phosphorites. The high Radioactivity of the studied phosphorites is probably due to the widespread occurrence of phosphatic components (e.g., apatite) that accommodate U in high concentrations. Gamma spectrometry based on NaI (Tl) crystal 3×3 has been used to measure occurring radionuclides in the phosphorite samples. The results indicate that the radioactive concentrations' average values of ^{226}Ra, ^{232}Th, and ^{40}K are 184.18±9.19, 125.82±6.29, and 63.82±3.19 Bq Kg^{-1}, respectively. Additionally, evaluations have been made of the radiological hazards. The calculated risk indicators exceeded the recommended national and world averages. The data obtained will serve as a reference for follow-up studies to evaluate the effectiveness of the Radioactivity of phosphatic materials collected from the Hamdat mine area.

1. Introduction

Radionuclides naturally spread throughout the earth because these nuclides are present in various geological materials such as rocks, soil, sand, water, coal, and phosphate deposits [1–3]. The Radioactivity in the scale samples comes from the ^{238}U to ^{232}Th series (and their decay progeny) as well as ^{40}K. These radioisotopes play a large role in a person's dose through external and internal exposure due to ingestion or inhalation [4-9]. Phosphates have many stable and radioactive elements that can be of environmental interest to the public [4]. The risks arise because of the release of dust and polluting residues into the environment from phosphate rock when used in industrial facilities, primarily in producing phosphoric acid and fertilizers [4]. It's worth looking at the elemental concentration in phosphate ores because of the rising use of phosphate in industries worldwide. It is used as a P-fertiliser and phosphorous source for chemical and food industries [5].

In Egypt, there is a wide occurrence of igneous, metamorphic, and sedimentary rocks which are concentric essentially in Sinai, the Eastern, and Western Deserts [6–14]. Phosphate deposits in Egypt are part of the Duwi Formation, which stretches from the Arab countries in Africa (to the west) to the Arab countries in Asia (to the east). According to [15], Egyptian phosphorite occurrences can be separated into three belts that tend in an east-west direction. The economic occurrences are only found in the central facies band and are confined to the following localities: the coast of the Red Sea from Safaga to Quseir; the Nile Valley between Idfu and Qena; and the Western Desert between the Kharga and Dakhla oases (Abu Tartur area). Age estimates [15–17] place the Phosphorite (Duwi) Formation somewhere in the range of the Upper Campanian to the Early Maastrichtian. This formation is conformably overlain by the Dakhla Formation.

Marine sedimentary phosphorites, the world's primary supply of phosphorus fertilizers, are formed intermittently along continental and oceanic basin margins. Egypt's phosphate resources are part of the Upper Cretaceous-Lower Paleogene Tethyan phosphonic belts that run through North Africa and the Middle East [18,19]. The economic ore beds are distributed along the Red Sea Coast, the Western Desert, and the Nile Valley. They originated in basins of intracratonic sediment in an epicontinental sea on the stable African shelf [20]. They were linked to the most promising black shale source rocks formed in a warm greenhouse climate [21]. The current work aims to measure the concentrations of (^{232}Th, ^{226}Ra, and ^{40}K) and radiological hazards for populations in samples of phosphates collected from the Hamadat mine area (Central Eastern Desert, Egypt). It is certain that the data extracted from this study is helpful for mapping natural radioactivity and can also be used as reference data for monitoring and identifying potential radioactive contamination in the future.

In this manuscript, we provide geochemical data, measured natural Radioactivity, and radiological risk parameters of the Hamadat mine phosphorites to study their geochemical characteristics and evaluate their radiological risk.

2. Materials and Methods

2.1. Whole-rock geochemical analysis

Phosphates were obtained in eleven samples from the Hamadat mining region (Central Eastern Desert, Egypt). The selected phosphorite samples were prepared for geochemical analysis. The samples were powdered using a mortar grinder in the Geology Department, Faculty of Science, Assiut University (Egypt). The major oxides (fused pellets) and trace elements (powder pellets) of the whole rock were measured using the Philips PW 24004, an X-Ray Fluorescence (XRF) technique at the University of Vienna (Austria). The details of the analytical steps were described by Ali et al.**[22]** The XRF analytical precision was estimated to be better than 0.5% for major oxides and 2% to 5% for trace elements.

2.2. Measurements and analysis using gamma spectrometry

The rest of the samples were crushed, homogenized, and sieved (200 m), which is the ideal size and enriched in heavy minerals after being dried at 110 ºC for 24 hours to remove the moisture content altogether. A 250 mL polyethene beaker served as the container for each sample. For four weeks, the beakers were sealed to establish secular equilibrium, which occurs when the daughters' decay rate reaches parity with that of the parents. This step's significance is to ensure that the daughters will remain in the sample and that the radon gas is contained within the volume[23].

The samples were examined using a high-resolution scintillation detector made of a 3-inch NaI (Tl) crystal in a gamma ray spectrometer. A 100 mm thick cylindrical lead shield with a fixed bottom and a moveable cover covered the detector to lower the gamma-ray background. Using reference sources such as ^{60}Co (1173.2 and 1332.5 keV), 133Ba (356.1 keV), and 137Cs (661.9 keV), the detection array's energy was calibrated. The IAEA-314 reference material, whose particular activity was known and contained the radionuclides ^{226}Ra, ^{232}Th, and ^{40}K, was used to create the efficiency calibration curve. An empty sealed beaker with the same geometry as the measured samples was placed around the detector to measure the background activity in order to adjust the net peak area of the detected isotopes. Twenty-eight thousand eight hundred seconds were used to measure the activity and the background. The average concentrations of ^{212}Pb (238.6 keV) and ^{228}Ac (911.1 keV) in the samples were used to calculate the concentration of ^{232}Th, and the average concentrations of the decay products of ^{214}Pb (351.9 keV) and ^{214}Bi (609.3 and 1764.5 keV) were used to calculate the concentration of ^{226}Ra. The 1461 keV peak was used to estimate the ^{40}K activity[24]. Referring to the IAEA reports, the lower limit of detection (LLD) is given by

$$\mathrm{LLD} = \frac{4.66\sqrt{F_c}}{\eta . P_\gamma . m . t} \qquad ((1)$$

where Fc is the Compton background in the region of the selected gamma line in the sample spectrum, ε is the system detection efficiency, Pγ is the absolute transition probability of gamma decay, m is the sample mass (in kilograms) and t is the counting time (in seconds). The lower limits of detection (LLD) for ^{226}Ra, ^{232}Th, and ^{40}K in samples are 2.4, 1.4, and 5.8 Bq kg^{-1}, respectively.

The activity concentration (A), the activity of radium equivalent (Ra_{eq}), absorbed dose rate (D), external and internal hazards indices (Hex, Hin), annual effective dose equivalent (AED), and excess lifetime cancer (ELCR), was calculated by the following equations[25–29]:

$$A = \frac{N_p}{e \times \eta \times m} \quad (2)$$

Where NP is the count per second, e is the abundance of the gamma peak in a radionuclide, η is the measured efficiency for each gamma peak observed for the same number of channels, either the sample or calibration source and m is the sample mass.

$$Raeq = ARa + (1.43ATh) + (0.077AK) \quad (3)$$

$$D = 0.427\ ARa + 0.662ATh + 0.0423\ AK \quad (4)$$

$$Hex = \frac{A_{Ra}}{370} + \frac{A_{Th}}{259} + \frac{A_K}{4810} \quad (5)$$

$$Hin = \frac{A_{Ra}}{185} + \frac{A_{Th}}{259} + \frac{A_K}{4810} \quad (6)$$

Where, ARa, ATh, and AK are the activities of ^{226}Ra, ^{232}Th, and ^{40}K, respectively, in Bq kg 1.

$$\text{Annual Effective Dose Rate} = D \times T \times F \quad (7)$$

Where, T is the outdoor occupation time (0.224 h365.25 d1753 hy^{-1}), D is the dose rate (in nGyh^{-1}), F is the conversion factor (0.7 10-6 SvGy-1),

$$ELCR = (AED) \times LD \times RF \quad (8)$$

Where DL is the life expectancy (70 years), and RF is the risk factor (Sv-1), which is 0.05. (ICRP-60).

3. Geologic background and petrography

In the Eastern Desert, the phosphate beds occur in the Duwi Formation. The Duwi Formation is overlain by laminated foraminiferal-rich marine shale and marl of the Late Formation of Maastrichtian Dakhla, which is conformably underlain by non-marine, the middle Campanian Quseir Formation-varicolored shale [30]. The Duwi deposits depict the first stage of marine transgression during the Late Cretaceous. These sediments indicate deposition in settings ranging from the inner neritic to the outer shelf, as well as recurrent sea-level variations [31]. Vertebrate remains, fish teeth, coprolites, heteromorph ammonites, and bivalves, as well as a few gastropods, echinoderms, baculitid ammonites, and nautiloid species, are recorded in this formation [32,33]. Based on the faunal assemblage in this formation, these deposits are mostly restricted to the Late Campanian-Early Maastrichtian time [34,35]. The Duwi Formation is occupied by alternating strata

of sandstone, claystone, siltstone, cherts, black shales, and oyster limestone that enclose a series of varying thicknesses of phosphatic interbed shales [30].

The Hamadat phosphatic mine area is bounded by longitudes 34°11′ 25″-34°11′ 32″E and latitudes 26°02′ 55″-26° 03′ 2″N in the Central Eastern Desert (Figure 1). The area can be easily reached through the Qift-Quseir asphaltic road about 16 km west. At the Hamadat mine area, the phosphates were primarily collected from the Duwi Formation horizontal strata (Figure 2), which extended to several kilometres and were deposited during late Cretaceous marine transgression events [34]. The phosphates occur in the form of beds that interact with shale, limestone, and chert.

The phosphorite samples are massive, fine- to medium-grained with grey, and yellow-to-brown colours (Figure 3 a-b). The studied phosphorites belong to the grain-supported type where the phosphatic components exceed 70 vol% of the rock. The phosphorite samples collected from the upper layers (1.5 m thickness) are grey and fossiliferous. At the same time, those collected from the medium and lower layers have common yellowish to brownish colours. They consist of phosphatic pellets, bioclasts (bones), non-phosphatic minerals, and cement (Figure 3 c-d). The isotropic phosphatic pellets represent a crypto-crystalline apatite (collophane) and occur as rounded to subrounded grains with dark and brown colours. These phosphatic pellets are partially replaced with cement materials such as calcedony and calcite. The anisotropic bioclasts are represented by angular to prismatic bone fragments and shells. The non-phosphatic materials are represented mainly by fine-grained quartz, mono- to crypto-crystalline chalcedony, calcite, and to a lesser extent, clay minerals. The cement/matrix is composed of cryptocrystalline calcareous and siliceous materials.

Figure 3. Various types of phosphate samples within the Hamadat Mine where a) shows the grey siliceous phosphorite and b) display the yellow calcareous phosphorite, c) photomicrographs show the rounded to subrounded phosphatic pellets (PP) surrounded by cryptocrystalline siliceous material, and d) bone fragments (BF), shells and phosphatic pellets impeded and surrounded with calcareous materials (CC).

4. Results and discussion

4.1. Geochemical characteristics of the Hamadat phosphorites

Whole rock major oxides along with some trace elements concentrations of representative phosphorite samples are provided in Table 1. The P_2O_5 content shows a narrow range that varies from 23.59 wt.% to 28.36 wt.%, with an average of 25.54 wt.%. While SiO2 shows a wide range that varies from 3.36 wt.% to 15.39

wt.%. The samples have a low concentration of Al_2O_3 (average = 0.37 wt.%) and TiO_2 (average = 0.03 wt.%). The Al_2O_3 concentration, which is the primary detrital component, has a typical inverse relationship with P_2O_5 (Figure 4a). Moreover, the samples have a low Fe_2O_3 (average = 1.77 wt.%) content, which is also negatively correlated with the P_2O_5 (Figure 4b). The samples have a narrow range of CaO concentrations (40.85-44.35 wt.%) and low Na_2O (average = 0.74 wt.%) content, which is positively correlated with P_2O_5 (Figure 4c). They also contain very low concentrations of K_2O (average = 0.74 wt.%) and MnO (average = 0.14 wt.%), suggesting the low contribution of the detrital material during the deposition of the studied phosphates.

The samples contain an extraordinarily high U concentration range from 55 to 128 ppm, with an average of 92 ppm, which means that they are uraniferous phosphorites formed at deep-water levels [36]. These high U concentrations are higher than those of the UCC (2.5 ppm;[37] and the PAAS (2.7 ppm; [38]. The samples contain high Sr (average = 1123 ppm), Ba (average = 87 ppm), Cr (average = 74 ppm), V (average = 87 ppm), and Zn (average = 242 ppm) concentrations, which could be due to biologic enrichment [39]. Th, Zr, and Rb occur at deficient concentrations (Table 1), which strongly supports the low detrital input during forming of the Hamadat phosphorites. The value of the U/Th ratio is high (15.28-21.43) and the content of U and Th is positively correlated with P_2O_5 (Figure 4d), which could be attributed to the substitution of U for Ca in the apatite structure. The high Sr/Ba values (12-16) of the studied phosphorites indicate that they were generated by normal marine sedimentation [39]. The wide occurrence of phosphatic components (e.g., apatite), that accommodate U with high concentrations is the main reason for the high Radioactivity of the studied phosphorites.

4.2. Activity concentrations and radiological parameters

Radioactive elements such as uranium phosphorite are available in phosphate rock. The presence of phosphate components that contain uranium is the leading cause of the radioactive abnormalities within and/or of their apatite minerals [40]. The ^{226}Ra, ^{232}Th, and ^{40}K concentrations in phosphate samples from the Hamadat mine area in units of Bq kg^{-1} are presented in Table 2. From Table 2, the activity concentration (Bq kg^{-1}) of ^{226}Ra ranged from 37.0±1.8 in the RP4 sample to 289±14.4 in the RP3 sample, with an average value of 184.18±9.19. For ^{232}Th the activity ranged from 7.00±0.3 in a sample coded by RP4 to 189±9.5 $Bqkg^{-1}$ in a sample coded by RP9 with an average value of 125.82±6.29. Finally, the activity of ^{40}K ranged from 30±1.5 in the sample code by RP2 to 123±6.1 in the sample code by RP3, with an average value of 63.82±3.19 $Bqkg^{-1}$. Phosphate samples coded by RP3 presented the highest activity for ^{226}Ra and ^{40}K, while phosphate samples coded by RP9 presented the highest activity for ^{232}Th. The lowest activities for ^{226}Ra, ^{232}Th and ^{40}K have been recorded in samples coded by Rb4 as shown in Figure 5.

Table 1. An example of a representative whole-rock geochemical study of the Hamadat Phosphorites

Sam-	RP	RP	RP	RP	RP	RP	RP	RP	RP	RP	RP1
P_2O_5	28.3	26.3	25.8	27.3	25.6	23.5	24.1	24.	24.	25.	25.1
SiO_2	3.36	4.25	4.49	4.71	4.86	15.3	15.2	14.	12.	10.	9.92
CaO	41.2	42.0	40.9	41.8	40.8	44.3	41.2	42.	40.	41.	42.6
Fe_2O_3	1.14	1.58	2.05	1.3	1.41	2.28	2.16	2.0	2	1.9	1.49
Al_2O_3	0.23	0.29	0.33	0.24	0.37	0.51	0.49	0.4	0.3	0.4	0.38
TiO_2	0.02	0.03	0.02	0.03	0.02	0.01	0.03	0.0	0.0	0.0	0.03
MnO	0.18	0.15	0.09	0.13	0.18	0.13	0.14	0.1	0.1	0.1	0.08
MgO	1.39	0.98	1.22	0.94	0.87	1.46	0.69	0.5	0.7	0.4	0.37
Na_2O	1.19	0.89	0.87	1.01	0.75	0.37	0.54	0.5	0.5	0.6	0.74
K_2O	0.03	0.04	0.03	0.03	0.05	0.07	0.12	0.1	0.0	0.1	0.09
Ba	114	102	95	106	91	57	69	76	79	82	84
Sr	131	123	121	125	121	896	948	998	104	110	112
Rb	5	12	16	9	7	6	11	4	8	13	10
Th	6.6	5.1	4.9	5.8	4.9	3.6	3.9	4.2	4.4	4.5	4.7
U	128	109	105	116	92	55	61	79	85	88	91
Zr	28	21	19	22	31	29	35	27	24	28	22
Cr	79	54	67	62	59	48	56	74	101	92	117
V	125	103	101	119	99	56	60	65	72	78	80
Ni	45	39	28	51	40	29	35	24	19	33	21
Zn	259	194	304	98	405	169	239	309	157	270	253
U/Th	19.3	21.3	21.4	20.0	18.7	15.2	15.6	18.	19.	19.	19.3
Sr/Ba	11.5	12.0	12.8	11.8	13.4	15.7	13.7	13.	13.	13.	13.3

Figure. 4. Binary relation between P_2O_5 and (a) Al_2O_3, (b) Fe_2O_3, (c) Na_2O, and (d) U + Th contents of the Hamadat phosphorites, where r is the calculated correlation coefficient.

According to the worldwide average concentrations (35, 35, and 400 Bqkg-1) for ^{226}Ra, ^{232}Th, and ^{40}K, respectively, which were reported by [25], our findings demonstrate that the samples' average value of ^{40}K

under investigation is lower than the world average, while the average value of ^{226}Ra and ^{232}Th are higher than the world average concentrations [3,41–45]. The variations in the activity concentration are associated with the methods by which the soils are concentrated as well as the radioactive distribution in the rocks from which they arise. The activity concentration of ^{226}Ra, ^{232}Th, and ^{40}K (Bq kg-1) in phosphate rocks compared to the data of similar studies from different countries are presented in Table 3.

Table 4 lists the values for the following parameters: radium equivalent activity (Ra_{eq}), dose rate (D), exterior and internal hazards indices (H_ex, H_{in}), annual effective dose (AED), and excess lifetime cancer risk (ELCR). Ra_{eq} values for the phosphate samples ranged from 80.8 to 474 Bq kg-1, with a mean value of 371.7 Bq kg-1, as shown in Table 3. These values are greater than the advised limit of 370 Bq kg-1.[26]. The dose rate values ranged from 22.6 to 207.7, with a mean value of 159.9 $nGyh^{-1}$, which is higher than the international limit of 59 $nGyh^{-1}$ [46]. As shown in columns 5 and 6 of Table 4, the mean values of external and internal hazards for phosphate samples are higher than the unity (permissible level) [25, 48–52], which causes harm to the populations in the region under investigation. On the other hand, the yearly effective dose values were more significant than the global average effective dose, about 70 μSvy^{-1}, and varied from 27.718 to 254.708 Svy^{-1} with a mean value of 196.1 Svy^{-1} [25]. Finally, the excess lifetime cancer risk values were lower than the global limit of 29 10-3, ranging from 9.70 E-05 to 8.91E-04.

Table 2: Radioelement activity concentrations (A) (^{226}Ra, ^{232}Th, and ^{40}K)

Region Name	Sample Code	A (Bq kg^{-1}) ^{226}Ra	A (Bq kg^{-1}) ^{232}Th	A (Bq kg-1) ^{40}K
Hamadat mine area	Rb1	286±14.3	127±6.4	68±3.4
	Rb2	174±8.7	158±7.9	30±1.5
	Rb 3	289±14.4	116±5.8	123±6.1
	Rb 4	37.0±1.8	7.00±0.3	49±2.5
	Rb 5	128±6.4	140±7.0	56±2.8
	Rb 6	263±13.1	117±5.8	63±3.1
	Rb 7	103±5.2	113±5.7	42±2.1
	Rb 8	127±6.3	139±6.9	55±2.8
	Rb 9	173±8.6	189±9.5	75±3.8
	Rb 10	251±12.5	101±5.0	107±5.3
	Rb 11	195±9.8	177±8.9	34±1.7

Average		184.18±9.19	125.82±6.29	63.82±3.19

Figure 5: ^{226}Ra, ^{232}Th, and ^{40}K Radioelement activity concentrations

Table 3: Phosphate rocks from a variety of countries were tested to determine the activity concentration of ^{226}Ra, ^{232}Th and ^{40}K ($Bq\ kg^{-1}$)

Country	A ($Bq\ kg^{-1}$) ^{226}Ra	A ($Bq\ kg^{-1}$) ^{232}Th	A (Bq kg-1) ^{40}K	References
Egypt (Abu-Tartur)	117.6	65	126	[52]
Egypt (El-Mahamid)	567	217.3	217.3	[53]
Egypt (W. El-Mashash)	666	329.4	329.4	[53]
Egypt (El-Sibaiya)	538	2.5	N.F	[54]
Egypt (El-Quseir)	358	38	N.F	[54]
Egypt (Abu-Zaabal)	214	37	19	[55]
Finland	10	10	110	[39]
Pakistan (Hazara)	440	50	207	[39]
Tanzania (Arusha)	5022	717	286	[53]
**Egypt(the Hamadat mine area)**	**184.18±9.19**	**125.82±6.29**	**63.82±3.19**	**This work**

Table 4: Annual effective dose (AED), radium equivalent activity (Ra_{eq}), dose rate (D), external hazards index (H_{ex}) and internal hazards index (Hin), and excess lifetime cancer risk (ELCR) were measured for the samples.

Region Name	Sample Code.	Ra_{eq} ($BqKg^{-1}$)	D ($nGyh^{-1}$)	H_{ex}	H_{in}	AED (μSvy^{-1})	ELCR
Ha m ad	Rb1	474.0	207.7	1.281	2.055	254.708	8.91E-04

Rb2	401.4	172.3	1.084	1.554	211.300	7.40E-04
Rb 3	464.4	204.5	1.255	2.035	250.798	8.78E-04
Rb 4	80.8	22.6	0.135	0.233	27.718	9.70E-05
Rb 5	332.8	142.4	0.899	1.245	174.694	6.11E-04
Rb 6	434.5	190.4	1.774	1.884	233.483	8.17E-04
Rb 7	268.5	114.9	0.725	1.004	140.901	4.93E-04
Rb 8	329.1	140.9	0.889	1.231	172.774	6.05E-04
Rb 9	449.2	192.3	1.213	1.680	235.836	8.25E-04
Rb 10	403.1	177.5	1.089	1.766	217.706	7.62E-04
Rb 11	451.3	193.7	1.219	1.747	237.599	8.32E-04
**Average**	**371.7**	**159.9**	**1.1**	**1.5**	**196.1**	**6.86E-04**

4.3. Statistical analysis

Table 5 displays the statistical characteristics of the data that were collected (including the lowest, median, range, mean, maximum, standard deviation, standard error, skewness, and kurtosis of the radionuclides for each of the phosphate samples). It is clear from looking at Table 5 that the mean activity concentrations do not correspond to one another in any way. The value of ^{40}K has a positive skewness, which indicates that the distribution it represents has an unbalanced tail that extends towards more positive values. On the other hand, a negative skewness indicates a distribution with an asymmetric tail that extends towards greater negative values, which is exactly what we see in the case of ^{226}Ra and ^{232}Th. In conclusion, ^{232}Th and ^{40}K both show a positive kurtosis, which indicates that their distributions are relatively peaky[41,56].

Figure 6 shows the correlation coefficients between the radionuclides ^{226}Ra, ^{232}Th, and ^{40}K and radiation hazard parameters at the Hamadat mine area. From figure 6 (A1, A2 and A3), we can see that ^{226}Ra showed strong positive correlations with hazard parameters (ELCR, AEDE, and D), while ^{40K} showed weak connections, as shown in figure 6 (B1, B2, and B3). This indicates that the ^{226}Ra was the essential reason for the hazard parameters.

Table 5: Descriptive statistics of ^{238}U, ^{232}Th and ^{40}K (Bq kg^{-1}) in the Hamadat mine area.

	^{226}Ra	^{232}Th	^{40}K
Number of values	11	11	11
Minimum	37	7	30

25% Percentile	127	113	42
Median	174	127	56
75% Percentile	263	158	75
Maximum	289	189	123
Range	252	182	93
Mean	184.2	125.8	63.82
Standard Deviation	81.89	48.06	28.94
Standard Error of Mean	24.69	14.49	8.727
Skewness	-0.2434	-1.4	1.055
Kurtosis	-0.8229	3.521	0.5727

Figure 6. Correlation coefficients illustrate the relation between the radionuclides ^{226}Ra, ^{232}Th, ^{40}K, and radiation hazard parameters at the Hamadat mine area.

Conclusions

The phosphorite samples were collected from the Duwi Formation late Cretaceous horizontal strata at the Hamadat phosphatic mine, which is located in the Central Eastern Desert of Egypt. The samples consist of bioclasts and crypto-crystalline apatite (collophane), which are partially replaced with cement materials (calcedony and calcite). The samples contain high P_2O_5, CaO, U, Sr, Ba, Cr, V and Zn concentrations, with a notably very low content of Al_2O_3, Fe_2O_3, Na_2O, K_2O, MnO, Th, Zr and Rb. The high Sr/Ba values indicate that phosphorites were generated by normal marine sedimentation. The high U/Th ratio could be attributed to the substitution of U for Ca in the apatite structure, which also increases the Radioactivity within the studied phosphorites. The spectrometer of Gamma-ray includes a high-scintillation detector of resolution NaI (Tl) crystal 3×3 has been used to measure occurring radionuclides in the studied phosphorites. The levels of natural Radioactivity in the samples are more significant than permitted. Additionally, the mean radiological risk values were higher than the world average, making it unsafe for the population living there. The information gathered here serves as a reference point for creating an environmental map of the Hamadat phosphate mine area while also showing that any potential effects of the mining operations if any exist, are minimal and obscured by variations in the background values that are inherent to nature.

Article

ORIGINALITY REPORT

24%	17%	23%	%
SIMILARITY INDEX	INTERNET SOURCES	PUBLICATIONS	STUDENT PAPERS

PRIMARY SOURCES

1	academic.oup.com<br>Internet Source	3%
2	link.springer.com<br>Internet Source	2%
3	Atef El-Taher, Fatimh Alshahri, Reda Elsaman. "Environmental impacts of heavy metals, rare earth elements and natural radionuclides in marine sediment from Ras Tanura, Saudi Arabia along the Arabian Gulf", Applied Radiation and Isotopes, 2018<br>Publication	2%
4	www.naturalspublishing.com<br>Internet Source	2%
5	cyberleninka.org<br>Internet Source	1%
6	El Saeed R. Lasheen, Hesham M. H. Zakaly, B. M. Alotaibi, Diaa A. Saadawi et al. "Radiological Risk Parameters of the Phosphorite Deposits, Gebel Qulu El Sabaya:	1%

Natural Radioactivity and Geochemical Characteristics", Minerals, 2022
Publication

7	ijrr.com<br>Internet Source	1 %
8	mdpi-res.com<br>Internet Source	1 %
9	"The Geology of the Egyptian Nubian Shield", Springer Science and Business Media LLC, 2021<br>Publication	1 %
10	M.A.M. Uosif, Shams A.M. Issa, L.M. Abd El-Salam. "Measurement of natural radioactivity in granites and its quartz-bearing gold at El-Fawakhir area (Central Eastern Desert), Egypt", Journal of Radiation Research and Applied Sciences, 2019<br>Publication	1 %
11	Eheb Massoud, Atef El-Taher, Laith Ahmed Najam, Reda Elsaman. "Ecological impacts of Assuit fertiliser factory in Upper Egypt: environmental implications and spatial distribution of natural radionuclides", International Journal of Environmental Analytical Chemistry, 2021<br>Publication	1 %

12	Reda Elsaman, Mohammed Ahmed Ali, El-Montaser Mahmoud Se, Atef El-Taher. "Natural Radioactivity Levels and Radiological Hazards in Soil Samples Around Abu Karqas Sugar Factory", Journal of Environmental Science and Technology, 2018<br>Publication	1%
13	Atef El-Taher, Hesham M.H. Zakaly, Reda Elsaman. "Environmental implications and spatial distribution of natural radionuclides and heavy metals in sediments from four harbours in the Egyptian Red Sea coast", Applied Radiation and Isotopes, 2018<br>Publication	1%
14	Awad A. Ibraheem, Atef El-Taher, May H.M. Alruwaili. "Assessment of natural radioactivity levels and radiation hazard indices for soil samples from Abha, Saudi Arabia", Results in Physics, 2018<br>Publication	1%
15	mafiadoc.com<br>Internet Source	1%
16	Xinwei Lu, Xiaolan Zhang. "Natural radioactivity and associated radiation hazards of river sands in Xi'an, China", Radiation Effects and Defects in Solids, 2009<br>Publication	<1%

17	Fawzia Ahmad, Hany A. Shousha, Hassan M. Diab. "Comparative study of natural radioactivity concentrations in soil samples from the newly developed Tushki and Giza Regions in Egypt", Radiation Effects and Defects in Solids, 2006<br>Publication	<1%
18	S. U. El-Kameesy, E. Salama, S. A. El-fiki, M. Ehab, W. Rühm. "Radiological safety assessment inside ancient Egyptian tombs in Saqqara", Isotopes in Environmental and Health Studies, 2016<br>Publication	<1%
19	Md. Joynal Abedin, Rahat Khan. "NORMs distribution in the dust samples from the educational institutions of Megacity Dhaka, Bangladesh: Radiological risk assessment", Journal of Hazardous Materials Advances, 2022<br>Publication	<1%
20	A. A. Orabi. "Natural radioactivity levels and radiation hazard indices in granite from Aswan to Wadi El-Allaqi southeastern desert, Egypt", Radiation Protection Dosimetry, 06/04/2007<br>Publication	<1%
21	Khan, K.. "Norm and associated radiation hazards in bricks fabricated in various	<1%

localities of the North-West Frontier Province (Pakistan)", Journal of Environmental Radioactivity, 2002
Publication

---

22 Lingqing Wang, Xinwei Lu. "Natural radionuclide concentrations in soils around Baoji coal-fired power plant, China", Radiation Effects and Defects in Solids, 2007 — <1%
Publication

---

23 Esmat A. Abou El-Anwar, Said H. Abd El Rahim. "Mineralogy, geochemistry and origin of the phosphorites at Um El-Huwtat mine, Quseir, Central Eastern Desert, Egypt", Carbonates and Evaporites, 2022 — <1%
Publication

---

24 es.scribd.com — <1%
Internet Source

---

25 Kucukomeroglu, B., F. Maksutoglu, N. Damla, U. Cevik, and N. Celebi. "A STUDY OF ENVIRONMENTAL RADIOACTIVITY MEASUREMENTS IN THE SAMSUN PROVINCE, TURKEY", Radiation Protection Dosimetry, 2012. — <1%
Publication

---

26 M A M Uosif, Z A Alrowaili, Reda Elsaman, A M A Mostafa. "SOIL–SOYBEAN TRANSFER FACTOR OF NATURAL RADIONUCLIDES IN DIFFERENT SOIL TEXTURES AND THE — <1%

ASSESSMENT OF COMMITTED EFFECTIVE DOSE", Radiation Protection Dosimetry, 2020
Publication

27	oar.icrisat.org<br>Internet Source	<1 %
28	Muneyuki Matsunaga, Hironori Nagano, Tetsuo Mikami, Tomohiko Kubo. "Large 3' UTR of sugar beet rps3 is truncated in cytoplasmic male-sterile mitochondria", Plant Cell Reports, 2010<br>Publication	<1 %
29	N.N. Jibiri. "Radiological hazard indices due to activity concentrations of natural radionuclides in farm soils from two high background radiation areas in Nigeria", International Journal of Low Radiation, 2009<br>Publication	<1 %
30	journals.yu.edu.jo<br>Internet Source	<1 %
31	El-Taher, Atef, and Hashem A. Madkour. "Environmental and radio-ecological studies on shallow marine sediments from harbour areas along the Red Sea coast of Egypt for identification of anthropogenic impacts", Isotopes in Environmental and Health Studies, 2014.<br>Publication	<1 %

32	G. Gurbuz. "Radiological significance of cement used in building construction in Turkey", Radiation Protection Dosimetry, 10/19/2007<br>Publication	<1 %
33	I. P. Farai. "Gamma activity and radiation dose in concrete building blocks used for construction of dwellings in Jos, Nigeria", Radiation Protection Dosimetry, 12/01/2006<br>Publication	<1 %
34	docksci.com<br>Internet Source	<1 %
35	Abbady, A.G.E.. "Natural radioactivity and dose assessment for phosphate rocks from Wadi El-Mashash and El-Mahamid Mines, Egypt", Journal of Environmental Radioactivity, 2005<br>Publication	<1 %
36	Coulibaly Vamoussa, Sei Joseph, Kouame N`Dri, Koua Aka Antonin, Oyetola Samuel, Brun Stephane. "Measurement of Natural Radioactivity in the Clays Consummated in Côte d’Ivoire using Gamma-ray Spectrometry", Journal of Applied Sciences, 2012<br>Publication	<1 %

37	Nuraddeen Nasiru Garba, Ahmad Termizi Ramli, Muneer Aziz Saleh, Hamman Tukur Gabdo. "Natural radioactivity and associated radiation hazards in soil of Kelantan, Malaysia", Human and Ecological Risk Assessment: An International Journal, 2018<br>Publication	<1 %
38	Atef El-Taher, Saleh Alashrah, Hashem A. Madkour, A. Al-Sayed, Taha M. El-Eria. "Radionuclides Distribution in Marine Sediment from Abu Soma Bay, Egyptian Red Sea Coast", Journal of Environmental Science and Technology, 2018<br>Publication	<1 %
39	CRAIG R. GLENN. "Anatomy and origin of a Cretaceous phosphorite-greensand giant, Egypt", Sedimentology, 2/1990<br>Publication	<1 %

Exclude quotes	On	Exclude matches	< 5 words
Exclude bibliography	On		

Article

GRADEMARK REPORT

FINAL GRADE

/0

GENERAL COMMENTS

Instructor

PAGE 1

PAGE 2

PAGE 3

PAGE 4

PAGE 5

PAGE 6

PAGE 7

PAGE 8

PAGE 9

PAGE 10

PAGE 11
