# Supplementary material for: Assessing geochemical and natural radioactivity impacts of Hamadat phosphatic mine through radiological indices
Source: PLoS One. 2023 Aug 3;18(8):e0287422. doi: 10.1371/journal.pone.0287422 (PMC10399901; doi:10.1371/journal.pone.0287422)
Supplement: Attachment — Submitted filename: Manuscript_ PONE-annotated.docx [file pone.0287422.s001.docx]

**Implications of radiological indices of Hamadat phosphatic mine: Geochemical and Natural radioactivity aspects**

Douaa Fathy^1^, Hesham M.H. Zakaly^2,3^*, B.M. Alotaibi^4^, El Saeed R. Lasheen^5^, Reda Elsaman^2^, Mabrouk Sami^1^, Hamdy A. Awad^6^, Antoaneta Ene^7^*

1 Geology Department, Faculty of Science, Minia University, El-Minia, 61519, Egypt;
2 Physics Department, Faculty of Science, Al-Azhar University, Assiut Branch, 71524 Egypt.
3 Institute of Physics and Technology, Ural Federal University, Yekaterinburg 620002, Russia
4 Department of Physics, College of Science, Princess Nourah Bint Abdulrahman University, P.O. Box 84428, Riyadh, 11671, Saudi Arabia;
5 Geology Department, Faculty of Science, Al-Azhar University, Cairo, P.O. Box 11884, Egypt;
6 Geology Department, Faculty of Science, Al-Azhar University, Assiut Branch, 71524 Egypt,
7 Dunarea de Jos University of Galati, INPOLDE Research Center, Faculty of Sciences and Environment, Department of Chemistry, Physics and Environment, 47 Domneasca Street, 800008 Galati, Romania;

*Corresponding; (Hesham M. Zakaly) [h.m.zakaly@azhar.edu.eg](mailto:h.m.zakaly@azhar.edu.eg), (Antoaneta Ene), [Antoaneta.Ene@ugal.ro](mailto:Antoaneta.Ene@ugal.ro)

**Abstract:** The present work manifests the geochemical characteristics, distribution of natural Radioactivity, and radiological risk assessment of the phosphorite deposits to assess their favorability in industrial applications such as preparing phosphoric acid and phosphatic fertilizers. The phosphorites are massive and collected from different beds within the Duwi Formation at the Hamadat mining area. They are grain-supported and composed of phosphatic pellets, bioclasts (bones), non-phosphatic minerals, and cement. Geochemically, phosphorites contain high concentrations of P_2_O_5_ (23.59- 28.36 wt.%) and CaO (40.85-44.35 wt.%), with low amounts of Al_2_O_3_, TiO_2_, Fe_2_O_3_, Na_2_O, K_2_O, and MnO, suggesting the low contribution of the detrital material during their deposition. Moreover, they belong to uraniferous phosphorites due to a notably high U concentration (55-128 ppm). They are also enriched with Sr, Ba, Cr, V, and Zn and depleted in Th, Zr, and Rb, which strongly supports the low detrital input during the formation of the Hamadat phosphorites. The high Radioactivity of the studied phosphorites is probably due to the widespread occurrence of phosphatic components (e.g., apatite) that accommodate U in high concentrations. Gamma spectrometry based on NaI (Tl) crystal 3×3 has been used to measure occurring radionuclides in the phosphorite samples. The results indicate that the radioactive concentrations' average values of ^226^Ra, ^232^Th, and ^40^K are 184.18±9.19, 125.82±6.29, and 63.82±3.19 Bq Kg^-1^_,_ respectively. Additionally, evaluations have been made of the radiological hazards. The calculated risk indicators exceeded the recommended national and world averages. The data obtained will serve as a reference for follow-up studies to evaluate the effectiveness of the Radioactivity of phosphatic materials collected from the Hamdat mine area.

**Keywords:** Hamadat mine, Phosphorite, Radiological parameters, Radioactivity

1. Introduction

Radionuclides naturally spread throughout the earth because these nuclides are present in various geological materials such as rocks, soil, sand, water, coal, and phosphate deposits [1–3]. The Radioactivity in the scale samples comes from the ^238^U to ^232^Th series (and their decay progeny) as well as ^40^K. These radioisotopes play a large role in a person's dose through external and internal exposure due to ingestion or inhalation [4-9]. Phosphates have many stable and radioactive elements that can be of environmental interest to the public [4]. The risks arise because of the release of dust and polluting residues into the environment from phosphate rock when used in industrial facilities, primarily in producing phosphoric acid and fertilizers [4]. It's worth looking at the elemental concentration in phosphate ores because of the rising use of phosphate in industries worldwide. It is used as a P-fertiliser and phosphorous source for chemical and food industries [5].

In Egypt, there is a wide occurrence of igneous, metamorphic, and sedimentary rocks which are concentric essentially in Sinai, the Eastern, and Western Deserts [6–14]. Phosphate deposits in Egypt are part of the Duwi Formation, which stretches from the Arab countries in Africa (to the west) to the Arab countries in Asia (to the east). According to [15], Egyptian phosphorite occurrences can be separated into three belts that tend in an east-west direction. The economic occurrences are only found in the central facies band and are confined to the following localities: the coast of the Red Sea from Safaga to Quseir; the Nile Valley between Idfu and Qena; and the Western Desert between the Kharga and Dakhla oases (Abu Tartur area). Age estimates [15–17] place the Phosphorite (Duwi) Formation somewhere in the range of the Upper Campanian to the Early Maastrichtian. This formation is conformably overlain by the Dakhla Formation.

Marine sedimentary phosphorites, the world's primary supply of phosphorus fertilizers, are formed intermittently along continental and oceanic basin margins. Egypt's phosphate resources are part of the Upper Cretaceous-Lower Paleogene Tethyan phosphonic belts that run through North Africa and the Middle East [18,19]. The economic ore beds are distributed along the Red Sea Coast, the Western Desert, and the Nile Valley. They originated in basins of intracratonic sediment in an epicontinental sea on the stable African shelf [20]. They were linked to the most promising black shale source rocks formed in a warm greenhouse climate [21]. The current work aims to measure the concentrations of (^232^Th, ^226^Ra, and ^40^K) and radiological hazards for populations in samples of phosphates collected from the Hamadat mine area (Central Eastern Desert, Egypt). It is certain that the data extracted from this study is helpful for mapping natural radioactivity and can also be used as reference data for monitoring and identifying potential radioactive contamination in the future.

In this manuscript, we provide geochemical data, measured natural Radioactivity, and radiological risk parameters of the Hamadat mine phosphorites to study their geochemical characteristics and evaluate their radiological risk.

2. Materials and Methods

2.1. Whole-rock geochemical analysis

Phosphates were obtained in eleven samples from the Hamadat mining region (Central Eastern Desert, Egypt). The selected phosphorite samples were prepared for geochemical analysis. The samples were powdered using a mortar grinder in the Geology Department, Faculty of Science, Assiut University (Egypt). The major oxides (fused pellets) and trace elements (powder pellets) of the whole rock were measured using the Philips PW 24004, an X-Ray Fluorescence (XRF) technique at the University of Vienna (Austria). The details of the analytical steps were described by Ali et al.[22]. The XRF analytical precision was estimated to be better than 0.5% for major oxides and 2% to 5% for trace elements.

2.2. Measurements and analysis using gamma spectrometry

The rest of the samples were crushed, homogenized, and sieved (200 m), which is the ideal size and enriched in heavy minerals after being dried at 110 ^o^C for 24 hours to remove the moisture content altogether. A 250 mL polyethene beaker served as the container for each sample. For four weeks, the beakers were sealed to establish secular equilibrium, which occurs when the daughters' decay rate reaches parity with that of the parents. This step's significance is to ensure that the daughters will remain in the sample and that the radon gas is contained within the volume[23].

The samples were examined using a high-resolution scintillation detector made of a 3-inch NaI (Tl) crystal in a gamma ray spectrometer. A 100 mm thick cylindrical lead shield with a fixed bottom and a moveable cover covered the detector to lower the gamma-ray background. Using reference sources such as ^60^Co (1173.2 and 1332.5 keV), 133Ba (356.1 keV), and 137Cs (661.9 keV), the detection array's energy was calibrated. The IAEA-314 reference material, whose particular activity was known and contained the radionuclides ^226^Ra, ^232^Th, and ^40^K, was used to create the efficiency calibration curve. An empty sealed beaker with the same geometry as the measured samples was placed around the detector to measure the background activity in order to adjust the net peak area of the detected isotopes. Twenty-eight thousand eight hundred seconds were used to measure the activity and the background. The average concentrations of ^212^Pb (238.6 keV) and ^228^Ac (911.1 keV) in the samples were used to calculate the concentration of ^232^Th, and the average concentrations of the decay products of ^214^Pb (351.9 keV) and ^214^Bi (609.3 and 1764.5 keV) were used to calculate the concentration of ^226^Ra. The 1461 keV peak was used to estimate the ^40^K activity[24]. Referring to the IAEA reports, the lower limit of detection (LLD) is given by

| $\text{LLD}=\frac{4.66\sqrt{F_{c}}}{\eta.P_{\gamma}.m.t}$ | ((1) |
| --- | --- |

where Fc is the Compton background in the region of the selected gamma line in the sample spectrum, ε is the system detection efficiency, Pγ is the absolute transition probability of gamma decay, m is the sample mass (in kilograms) and t is the counting time (in seconds). The lower limits of detection (LLD) for ^226^Ra, ^232^Th, and ^40^K in samples are 2.4, 1.4, and 5.8 Bq kg^-1^, respectively.

The activity concentration (A), the activity of radium equivalent (Ra_eq_), absorbed dose rate (D), external and internal hazards indices (Hex, Hin), annual effective dose equivalent (AED), and excess lifetime cancer (ELCR), was calculated by the following equations[25–29]:

$A=\frac{N_{p}}{e\times\eta\times m}$	(2)

Where NP is the count per second, e is the abundance of the gamma peak in a radionuclide, η is the measured efficiency for each gamma peak observed for the same number of channels, either the sample or calibration source and m is the sample mass.

Raeq = ARa + (1.43ATh) + (0.077AK)	(3)

D = 0.427 ARa + 0.662ATh + 0.0423 AK	(4)

Hex = $\frac{A_{Ra}}{370}$ + $\frac{A_{Th}}{259}$ + $\frac{A_{K}}{4810}$	(5)

Hin = $\frac{A_{Ra}}{185}$ + $\frac{A_{Th}}{259}$ + $\frac{A_{K}}{4810}$	(6)

Where, ARa, ATh, and AK are the activities of ^226^Ra, ^232^Th, and ^40^K, respectively, in Bq kg 1.

Annual Effective Dose Rate = D×T×F	(7)

Where, T is the outdoor occupation time (0.224 h365.25 d1753 hy^-1^), D is the dose rate (in nGyh^-1^), F is the conversion factor (0.7 10-6 SvGy-1),

ELCR = (AED) × LD × RF	(8)

Where DL is the life expectancy (70 years), and RF is the risk factor (Sv-1), which is 0.05. (ICRP-60).

3. Geologic background and petrography

In the Eastern Desert, the phosphate beds occur in the Duwi Formation. The Duwi Formation is overlain by laminated foraminiferal-rich marine shale and marl of the Late Formation of Maastrichtian Dakhla, which is conformably underlain by non-marine, the middle Campanian Quseir Formation-varicolored shale [30]. The Duwi deposits depict the first stage of marine transgression during the Late Cretaceous. These sediments indicate deposition in settings ranging from the inner neritic to the outer shelf, as well as recurrent sea-level variations [31]. Vertebrate remains, fish teeth, coprolites, heteromorph ammonites, and bivalves, as well as a few gastropods, echinoderms, baculitid ammonites, and nautiloid species, are recorded in this formation [32,33]. Based on the faunal assemblage in this formation, these deposits are mostly restricted to the Late Campanian-Early Maastrichtian time [34,35]. The Duwi Formation is occupied by alternating strata of sandstone, claystone, siltstone, cherts, black shales, and oyster limestone that enclose a series of varying thicknesses of phosphatic interbed shales [30].

The Hamadat phosphatic mine area is bounded by longitudes 34°11′ 25′′-34°11′ 32′′E and latitudes 26°02′ 55′′-26° 03′ 2′′N in the Central Eastern Desert (Figure 1). The area can be easily reached through the Qift-Quseir asphaltic road about 16 km west. At the Hamadat mine area, the phosphates were primarily collected from the Duwi Formation horizontal strata (Figure 2), which extended to several kilometres and were deposited during late Cretaceous marine transgression events [34]. The phosphates occur in the form of beds that interact with shale, limestone, and chert.

**Figure 1**: Location map for the investigated Hamadat area (yellow star) in the Eastern Desert.

**Figure 2**. Lithostratigraphic column for the studied area [21].

The phosphorite samples are massive, fine- to medium-grained with grey, and yellow-to-brown colours (Figure 3 a-b). The studied phosphorites belong to the grain-supported type where the phosphatic components exceed 70 vol% of the rock. The phosphorite samples collected from the upper layers (1.5 m thickness) are grey and fossiliferous. At the same time, those collected from the medium and lower layers have common yellowish to brownish colours. They consist of phosphatic pellets, bioclasts (bones), non-phosphatic minerals, and cement (Figure 3 c-d). The isotropic phosphatic pellets represent a crypto-crystalline apatite (collophane) and occur as rounded to subrounded grains with dark and brown colours. These phosphatic pellets are partially replaced with cement materials such as calcedony and calcite. The anisotropic bioclasts are represented by angular to prismatic bone fragments and shells. The non-phosphatic materials are represented mainly by fine-grained quartz, mono- to crypto-crystalline chalcedony, calcite, and to a lesser extent, clay minerals. The cement/matrix is composed of cryptocrystalline calcareous and siliceous materials.

**Figure 3**. Various types of phosphate samples within the Hamadat Mine where a) shows the grey siliceous phosphorite and b) display the yellow calcareous phosphorite, c) photomicrographs show the rounded to subrounded phosphatic pellets (PP) surrounded by cryptocrystalline siliceous material, and d) bone fragments (BF), shells and phosphatic pellets impeded and surrounded with calcareous materials (CC).

4. Results and discussion
   4.1. Geochemical characteristics of the Hamadat phosphorites

Whole rock major oxides along with some trace elements concentrations of representative phosphorite samples are provided in Table 1. The P_2_O_5_ content shows a narrow range that varies from 23.59 wt.% to 28.36 wt.%, with an average of 25.54 wt.%. While SiO_2_ shows a wide range that varies from 3.36 wt.% to 15.39 wt.%. The samples have a low concentration of Al_2_O_3_ (average = 0.37 wt.%) and TiO_2_ (average = 0.03 wt.%). The Al_2_O_3_ concentration, which is the primary detrital component, has a typical inverse relationship with P_2_O_5_ (Figure 4a). Moreover, the samples have a low Fe_2_O_3_ (average = 1.77 wt.%) content, which is also negatively correlated with the P_2_O_5_ (Figure 4b). The samples have a narrow range of CaO concentrations (40.85-44.35 wt.%) and low Na_2_O (average = 0.74 wt.%) content, which is positively correlated with P_2_O_5_ (Figure 4c). They also contain very low concentrations of K_2_O (average = 0.74 wt.%) and MnO (average = 0.14 wt.%), suggesting the low contribution of the detrital material during the deposition of the studied phosphates.

The samples contain an extraordinarily high U concentration range from 55 to 128 ppm, with an average of 92 ppm, which means that they are uraniferous phosphorites formed at deep-water levels [36]. These high U concentrations are higher than those of the UCC (2.5 ppm;[37] and the PAAS 2.7 ppm; [38]. The samples contain high Sr (average = 1123± ppm), Ba (average = 87 ppm), Cr (average = 74 ppm), V (average = 87 ppm), and Zn (average = 242 ppm) concentrations, which could be due to biologic enrichment [39]. Th, Zr, and Rb occur at deficient concentrations (Table 1), which strongly supports the low detrital input during forming of the Hamadat phosphorites. The value of the U/Th ratio is high (15.28-21.43) and the content of U and Th is positively correlated with P_2_O_5_ (Figure 4d), which could be attributed to the substitution of U for Ca in the apatite structure. The high Sr/Ba values (12-16) of the studied phosphorites indicate that they were generated by normal marine sedimentation [39]. The wide occurrence of phosphatic components (e.g., apatite), that accommodate U with high concentrations is the main reason for the high Radioactivity of the studied phosphorites.

4.2. Activity concentrations and radiological parameters

Radioactive elements such as uranium phosphorite are available in phosphate rock. The presence of phosphate components that contain uranium is the leading cause of the radioactive abnormalities within and/or of their apatite minerals [40]. The ^226^Ra, ^232^Th, and ^40^K concentrations in phosphate samples from the Hamadat mine area in units of Bq kg^-1^ are presented in Table 2. From Table 2, the activity concentration (Bq kg^-1^) of ^226^Ra ranged from 37.0±1.8 in the RP4 sample to 289±14.4 in the RP3 sample, with an average value of 184.18±9.19. For ^232^Th the activity ranged from 7.00±0.3 in a sample coded by RP4 to 189±9.5 Bqkg^-1^ in a sample coded by RP9 with an average value of 125.82±6.29. Finally, the activity of ^40^K ranged from 30±1.5 in the sample code by RP2 to 123±6.1 in the sample code by RP3, with an average value of 63.82±3.19 Bqkg^-1^. Phosphate sample of RP3 presented the highest activity for ^226^Ra and ^40^K, while phosphate sample of RP9 presented the highest activity for ^232^Th. The lowest activities for ^226^Ra, ^232^Th and ^40^K have been recorded in samples coded by Rb4 as shown in Figure 5.

| **Table 1.** An example of a representative whole-rock geochemical study of the Hamadat Phosphorites that were analyzed (Central Eastern Desert, Egypt). | | | | | | | | | | | |
| --- | --- | --- | --- | --- | --- | --- | --- | --- | --- | --- | --- |
| **Sample** | **RP1** | **RP2** | **RP3** | **RP4** | **RP5** | **RP6** | **RP7** | **RP8** | **RP9** | **RP10** | **RP11** |
| **P_2_O_5_** | 28.36 | 26.35 | 25.84 | 27.31 | 25.66 | 23.59 | 24.15 | 24.59 | 24.89 | 25.02 | 25.19 |
| **SiO_2_** | 3.36 | 4.25 | 4.49 | 4.71 | 4.86 | 15.39 | 15.26 | 14.19 | 12.58 | 10.84 | 9.92 |
| **CaO** | 41.21 | 42.04 | 40.94 | 41.81 | 40.85 | 44.35 | 41.25 | 42.91 | 40.97 | 41.19 | 42.61 |
| **Fe_2_O_3_** | 1.14 | 1.58 | 2.05 | 1.3 | 1.41 | 2.28 | 2.16 | 2.08 | 2 | 1.94 | 1.49 |
| **Al_2_O_3_** | 0.23 | 0.29 | 0.33 | 0.24 | 0.37 | 0.51 | 0.49 | 0.44 | 0.39 | 0.42 | 0.38 |
| **TiO_2_** | 0.02 | 0.03 | 0.02 | 0.03 | 0.02 | 0.01 | 0.03 | 0.02 | 0.03 | 0.05 | 0.03 |
| **MnO** | 0.18 | 0.15 | 0.09 | 0.13 | 0.18 | 0.13 | 0.14 | 0.18 | 0.11 | 0.13 | 0.08 |
| **MgO** | 1.39 | 0.98 | 1.22 | 0.94 | 0.87 | 1.46 | 0.69 | 0.57 | 0.74 | 0.42 | 0.37 |
| **Na_2_O** | 1.19 | 0.89 | 0.87 | 1.01 | 0.75 | 0.37 | 0.54 | 0.57 | 0.59 | 0.62 | 0.74 |
| **K_2_O** | 0.03 | 0.04 | 0.03 | 0.03 | 0.05 | 0.07 | 0.12 | 0.15 | 0.07 | 0.14 | 0.09 |
| **Ba** | 114 | 102 | 95 | 106 | 91 | 57 | 69 | 76 | 79 | 82 | 84 |
| **Sr** | 1316 | 1231 | 1219 | 1258 | 1219 | 896 | 948 | 998 | 1048 | 1101 | 1121 |
| **Rb** | 5 | 12 | 16 | 9 | 7 | 6 | 11 | 4 | 8 | 13 | 10 |
| **Th** | 6.6 | 5.1 | 4.9 | 5.8 | 4.9 | 3.6 | 3.9 | 4.2 | 4.4 | 4.5 | 4.7 |
| **U** | 128 | 109 | 105 | 116 | 92 | 55 | 61 | 79 | 85 | 88 | 91 |
| **Zr** | 28 | 21 | 19 | 22 | 31 | 29 | 35 | 27 | 24 | 28 | 22 |
| **Cr** | 79 | 54 | 67 | 62 | 59 | 48 | 56 | 74 | 101 | 92 | 117 |
| **V** | 125 | 103 | 101 | 119 | 99 | 56 | 60 | 65 | 72 | 78 | 80 |
| **Ni** | 45 | 39 | 28 | 51 | 40 | 29 | 35 | 24 | 19 | 33 | 21 |
| **Zn** | 259 | 194 | 304 | 98 | 405 | 169 | 239 | 309 | 157 | 270 | 253 |
| **U/Th** | 19.39 | 21.37 | 21.43 | 20.00 | 18.78 | 15.28 | 15.64 | 18.81 | 19.32 | 19.56 | 19.36 |
| **Sr/Ba** | 11.54 | 12.07 | 12.83 | 11.87 | 13.40 | 15.72 | 13.74 | 13.13 | 13.27 | 13.43 | 13.35 |

Figure. 4. Binary relation between P_2_O_5_ and (a) Al_2_O_3_, (b) Fe_2_O_3_, (c) Na_2_O, and (d) U + Th contents of the Hamadat phosphorites, where r is the calculated correlation coefficient.

According to the worldwide average concentrations (35, 35, and 400 Bqkg-1) for ^226^Ra, ^232^Th, and ^40^K, respectively, which were reported by [25], our findings demonstrate that the samples' average value of ^40^K under investigation is lower than the world average, while the average value of ^226^Ra and ^232^Th are higher than the world average concentrations [3,41–45]. The variations in the activity concentration are associated with the methods by which the soils are concentrated as well as the radioactive distribution in the rocks from which they arise. The activity concentration of ^226^Ra, ^232^Th, and ^40^K (Bq kg-1) in phosphate rocks compared to the data of similar studies from different countries are presented in Table 3.

Table 4 lists the values for the following parameters: radium equivalent activity (Ra_eq_), dose rate (D), exterior and internal hazards indices (H_e_x, H_in_), annual effective dose (AED), and excess lifetime cancer risk (ELCR). Ra_eq_ values for the phosphate samples ranged from 80.8 to 474 Bq kg-1, with a mean value of 371.7 Bq kg-1, as shown in Table 3. These values are greater than the advised limit of 370 Bq kg-1.[26].

The dose rate values ranged from 22.6 to 207.7, with a mean value of 159.9 nGyh^-1^, which is higher than the international limit of 59 nGyh^-1^ [46]. As shown in columns 5 and 6 of Table 4, the mean values of external and internal hazards for phosphate samples are higher than the unity (permissible level) [25, 48–52], which causes harm to the populations in the region under investigation. On the other hand, the yearly effective dose values were more significant than the global average effective dose, about 70 μSvy^-1^, and varied from 27.718 to 254.708 Svy^-1^ with a mean value of 196.1 Svy^-1^ [25]. Finally, the excess lifetime cancer risk values were lower than the global limit of 29 10-3, ranging from 9.70 E-05 to 8.91E-04.

**Table 2: Radioelement activity concentrations (A) (^226^Ra, ^232^Th, and ^40^K)**

| **Region Name** | **Samples** | **A (Bq kg^-1^)**  **^226^Ra** | **A (Bq kg^-1^)**  **^232^Th** | **A (Bq kg-1)**  **^40^K** |
| --- | --- | --- | --- | --- |
| **Hamadat mine area** | Rb1 | 286±14.3 | 127±6.4 | 68±3.4 |
|  | Rb2 | 174±8.7 | 158±7.9 | 30±1.5 |
|  | Rb 3 | 289±14.4 | 116±5.8 | 123±6.1 |
|  | Rb 4 | 37.0±1.8 | 7.00±0.3 | 49±2.5 |
|  | Rb 5 | 128±6.4 | 140±7.0 | 56±2.8 |
|  | Rb 6 | 263±13.1 | 117±5.8 | 63±3.1 |
|  | Rb 7 | 103**±**5.2 | 113**±**5.7 | 42**±**2.1 |
|  | Rb 8 | 127**±**6.3 | 139**±**6.9 | 55**±**2.8 |
|  | Rb 9 | 173**±**8.6 | 189**±**9.5 | 75**±**3.8 |
|  | Rb 10 | 251±12.5 | 101±5.0 | 107±5.3 |
|  | Rb 11 | 195±9.8 | 177±8.9 | 34±1.7 |
| **Average** |  | **184.18±9.19** | **125.82±6.29** | **63.82±3.19** |

**­­­­**

**Figure 5:** ^226^Ra, ^232^Th, and ^40^K Radioelement activity concentrations

**Table 3: Phosphate rocks from a variety of countries were tested to determine the activity concentration of ^226^Ra, ^232^Th and ^40^K (Bq kg^-1^)**

| **Country** | **A (Bq kg^-1^)**  **^226^Ra** | **A (Bq kg^-1^)**  **^232^Th** | **A (Bq kg-1)**  **^40^K** | **References** |
| --- | --- | --- | --- | --- |
| Egypt (Abu-Tartur) | 117.6 | 65 | 126 | [52] |
| Egypt (El-Mahamid) | 567 | 217.3 | 217.3 | [53] |
| Egypt (W. El-Mashash) | 666 | 329.4 | 329.4 | [53] |
| Egypt (El-Sibaiya) | 538 | 2.5 | N.F | [54] |
| Egypt (El-Quseir) | 358 | 38 | N.F | [54] |
| Egypt (Abu-Zaabal) | 214 | 37 | 19 | [55] |
| Finland | 10 | 10 | 110 | [39] |
| Pakistan (Hazara) | 440 | 50 | 207 | [39] |
| Tanzania (Arusha) | 5022 | 717 | 286 | [53] |
| **Egypt(the Hamadat mine area)** | **184.18±9.19** | **125.82±6.29** | **63.82±3.19** | **This work** |

**Table 4:** Annual effective dose (AED), radium equivalent activity (Ra_eq_), dose rate (D), external hazards index (H_ex_) and internal hazards index (Hin), and excess lifetime cancer risk (ELCR) were measured for the samples.

| **Region Name** | **Samples** | **Ra_eq_** | **D** | **H_ex_** | **H_in_** | **AED** | **ELCR** |
| --- | --- | --- | --- | --- | --- | --- | --- |
|  |  | **(BqKg^-1^)** | **(nGyh^-1^)** |  |  | **(μSvy^-1^)** |  |
| **Hamadat mine area** | Rb1 | 474.0 | 207.7 | 1.281 | 2.055 | 254.708 | 8.91E-04 |
|  | Rb2 | 401.4 | 172.3 | 1.084 | 1.554 | 211.300 | 7.40E-04 |
|  | Rb 3 | 464.4 | 204.5 | 1.255 | 2.035 | 250.798 | 8.78E-04 |
|  | Rb 4 | 80.8 | 22.6 | 0.135 | 0.233 | 27.718 | 9.70E-05 |
|  | Rb 5 | 332.8 | 142.4 | 0.899 | 1.245 | 174.694 | 6.11E-04 |
|  | Rb 6 | 434.5 | 190.4 | 1.774 | 1.884 | 233.483 | 8.17E-04 |
|  | Rb 7 | 268.5 | 114.9 | 0.725 | 1.004 | 140.901 | 4.93E-04 |
|  | Rb 8 | 329.1 | 140.9 | 0.889 | 1.231 | 172.774 | 6.05E-04 |
|  | Rb 9 | 449.2 | 192.3 | 1.213 | 1.680 | 235.836 | 8.25E-04 |
|  | Rb 10 | 403.1 | 177.5 | 1.089 | 1.766 | 217.706 | 7.62E-04 |
|  | Rb 11 | 451.3 | 193.7 | 1.219 | 1.747 | 237.599 | 8.32E-04 |
| **Average** | | **371.7** | **159.9** | **1.1** | **1.5** | **196.1** | **6.86E-04** |

**4.3. Statistical analysis**

Table 5 displays the statistical characteristics of the data that were collected (including the lowest, median, range, mean, maximum, standard deviation, standard error, skewness, and kurtosis of the radionuclides for each of the phosphate samples). It is clear from looking at Table 5 that the mean activity concentrations do not correspond to one another in any way. The value of ^40^K has a positive skewness, which indicates that the distribution it represents has an unbalanced tail that extends towards more positive values. On the other hand, a negative skewness indicates a distribution with an asymmetric tail that extends towards greater negative values, which is exactly what we see in the case of ^226^Ra and ^232^Th. In conclusion, ^232^Th and ^40^K both show a positive kurtosis, which indicates that their distributions are relatively peaky[41,56].

Figure 6 shows the correlation coefficients between the radionuclides ^226^Ra, ^232^Th, and ^40^K and radiation hazard parameters at the Hamadat mine area. From figure 6 (A1, A2 and A3), we can see that ^226^Ra showed strong positive correlations with hazard parameters (ELCR, AEDE, and D), while ^40K^ showed weak connections, as shown in figure 6 (B1, B2, and B3). This indicates that the ^226^Ra was the essential reason for the hazard parameters.

**Table 5:** Descriptive statistics of ^238^U, ^232^Th and ^40^K (Bq kg^-1^) in the Hamadat mine area.

|  | ^226^Ra | ^232^Th | ^40^K |
| --- | --- | --- | --- |
| Number of values | 11 | 11 | 11 |
|  |  |  |  |
| Minimum | 37 | 7 | 30 |
| 25% Percentile | 127 | 113 | 42 |
| Median | 174 | 127 | 56 |
| 75% Percentile | 263 | 158 | 75 |
| Maximum | 289 | 189 | 123 |
| Range | 252 | 182 | 93 |
|  |  |  |  |
| Mean | 184.2 | 125.8 | 63.82 |
| Standard Deviation | 81.89 | 48.06 | 28.94 |
| Standard Error of Mean | 24.69 | 14.49 | 8.727 |
|  |  |  |  |
| Skewness | -0.2434 | -1.4 | 1.055 |
| Kurtosis | -0.8229 | 3.521 | 0.5727 |

**Figure 6.** Correlation coefficients illustrate the relation between the radionuclides ^226^Ra, ^232^Th, ^40^K, and radiation hazard parameters at the Hamadat mine area.

Conclusions

The phosphorite samples were collected from the Duwi Formation late Cretaceous horizontal strata at the Hamadat phosphatic mine, which is located in the Central Eastern Desert of Egypt. The samples consist of bioclasts and crypto-crystalline apatite (collophane), which are partially replaced with cement materials (calcedony and calcite). The samples contain high P_2_O_5_, CaO, U, Sr, Ba, Cr, V and Zn concentrations, with a notably very low content of Al_2_O_3_, Fe_2_O_3_, Na_2_O, K_2_O, MnO, Th, Zr and Rb. The high Sr/Ba values indicate that phosphorites were generated by normal marine sedimentation. The high U/Th ratio could be attributed to the substitution of U for Ca in the apatite structure, which also increases the Radioactivity within the studied phosphorites. The spectrometer of Gamma-ray includes a high-scintillation detector of resolution NaI (Tl) crystal 3×3 has been used to measure occurring radionuclides in the studied phosphorites. The levels of natural Radioactivity in the samples are more significant than permitted. Additionally, the mean radiological risk values were higher than the world average, making it unsafe for the population living there. The information gathered here serves as a reference point for creating an environmental map of the Hamadat phosphate mine area while also showing that any potential effects of the mining operations if any exist, are minimal and obscured by variations in the background values that are inherent to nature.

**Funding**: The APC was covered by “Dunarea de Jos” University of Galati, Romania.

**Acknowledgments:** none.

**Data Availability Statement:** The data presented in this study are available on request from the corresponding authors.

**Conflicts of Interest:** The authors declare no conflict of interest.

References

1.	Elsaman, R.; Ali, G.; Uosif, M. A. M., Shaaban, K. H. S., Saddeek, Y. B., Aly, K. Chong, F. Natural Radioactivity of Some Egyptian Materials Used in Glasses Manufacturing and Glass Ceramics. Int J Radiat Res. 2018; 16 (2) :207-215

2.	Dugalic, G.; Krstic, D.; Jelic, M.; [Nikezic](https://pubmed.ncbi.nlm.nih.gov/?term=Nikezic+D&cauthor_id=20060645),D., [Milenkovic](https://pubmed.ncbi.nlm.nih.gov/?term=Milenkovic+B&cauthor_id=20060645), B., [Pucarevic](https://pubmed.ncbi.nlm.nih.gov/?term=Pucarevic+M&cauthor_id=20060645), M., [Zeremski-Skoric](https://pubmed.ncbi.nlm.nih.gov/?term=Zeremski-Skoric+T&cauthor_id=20060645), T. Heavy Metals, Organics and Radioactivity in Soil of Western Serbia. J. Hazard Mater. **2010** 15;177(1-3):697-702.

3.	Abbasi, A.; Zakaly, H.M.H.; Algethami, M.; Abdel-Hafez, S.H. Radiological Risk Assessment of Natural Radionuclides in the Marine Ecosystem of the Northwest Mediterranean Sea. *Int. J. Radiat. Biol.* **2022**, *98*, 205–211, doi:10.1080/09553002.2022.2020359.

4.	El-Bahi, S.M.; Sroor, A.; Mohamed, G.Y.; El-Gendy, N.S. Radiological Impact of Natural Radioactivity in Egyptian Phosphate Rocks, Phosphogypsum and Phosphate Fertilizers. *Appl. Radiat. Isot.* **2017**, *123*, 121–127, doi:10.1016/j.apradiso.2017.02.031.

5.	GLENN, C.R.; ARTHUR, M.A. Anatomy and Origin of a Cretaceous Phosphorite‐greensand Giant, Egypt. *Sedimentology* **1990**, *37*, 123–154, doi:10.1111/J.1365-3091.1990.TB01986.X.

6.	Abdel-Karim, A.-A.; Azer, M.; Sami, M. Petrogenesis and Tectonic Implications of the Maladob Ring Complex in the South Eastern Desert, Egypt: New Insights from Mineral Chemistry and Whole-Rock Geochemistry. *Int. J. Earth Sci.* **2021**, *110*, 53–80, doi:10.1007/s00531-020-01937-2.

7.	Hamdy, M.M.; Lasheen, E.S.R.; Abdelwahab, W. Gold-Bearing Listwaenites in Ophiolitic Ultramafics from the Eastern Desert of Egypt: Subduction Zone-Related Alteration of Neoproterozoic Mantle? *J. Afr. Earth Sci.* **2022**, *193*, 104574, doi:10.1016/j.jafrearsci.2022.104574.

8.	Khaleal, F.M.; Saleh, G.M.; Lasheen, E.S.R.; Lentz, D.R. Occurrences and Genesis of Emerald and Others Beryl Mineralization in Egypt: A Review. *Phys. Chem. Earth Parts ABC* **2022**, 103266, doi:10.1016/j.pce.2022.103266.

9.	Khaleal, F.M.; Saleh, G.M.; Lasheen, E.S.R.; Alzahrani, A.M.; Kamh, S.Z. Exploration and Petrogenesis of Corundum-Bearing Pegmatites: A Case Study in Migif-Hafafit Area, Egypt. *Front. Earth Sci.* **2022**, *10*, 869828, doi:10.3389/feart.2022.869828.

10.	Lasheen, E.S.R.; Mohamed, W.H.; Ene, A.; Awad, H.A.; Azer, M.K. Implementation of Petrographical and Aeromagnetic Data to Determine Depth and Structural Trend of Homrit Waggat Area, Central Eastern Desert, Egypt. *Appl. Sci.* **2022**, *12*, 8782, doi:10.3390/app12178782.

11.	Lasheen, E.S.R.; Saleh, G.M.; Khaleal, F.M.; Alwetaishi, M. Petrogenesis of Neoproterozoic Ultramafic Rocks, Wadi Ibib&ndash;Wadi Shani, South Eastern Desert, Egypt: Constraints from Whole Rock and Mineral Chemistry. *Appl. Sci. 2021 Vol 11 Page 10524* **2021**, *11*, 10524, doi:10.3390/APP112210524.

12.	Saleh, G.M.; Khaleal, F.M.; Lasheen, E.S.R. Geochemistry and Paleoweathering of Metasediments and Pyrite-Bearing Quartzite during the Neoproterozoic Era, Wadi Ibib-Wadi Suwawrib, South Eastern Desert, Egypt. *Arab. J. Geosci.* **2022**, *15*, 51, doi:10.1007/s12517-021-09141-5.

13.	Saleh, G.M.; Khaleal, F.M.; Lasheen, E.S.R. Petrogenesis of Ilmenite-Bearing Mafic Intrusions: A Case Study of Abu Ghalaga Area, South Eastern Desert, Egypt. *Arab. J. Geosci.* **2022**, *15*, 1508, doi:10.1007/s12517-022-10782-3.

14.	Saleh, G.M.; Kamar, M.S.; Lasheen, E.S.R.; Ibrahim, I.H.; Azer, M.K. Whole Rock and Mineral Chemistry of the Rare Metals-Bearing Mylonitic Rocks, Abu Rusheid Borehole, South Eastern Desert, Egypt. *J. Afr. Earth Sci.* **2022**, 104736, doi:10.1016/j.jafrearsci.2022.104736.

15.	Hermina, M.H. "Review on the Phosphate Deposits of Egypt. In Proceedings of the Proceedings of the second Arab conference of mineral resources.; 1972.

16.	Issawi, B.; Hassan, M.Y.; Saad, E.N. Geology of Abu Tartur Plateau, Western Desert, Egypt. *Ann Geol Surv Egypt* **1978**, *8*, 91-127.

17.	Issawi, B.; El Hinnawi, M.; Francis, M.; Mazhar, A. The Phanerozoic Geology of Egypt. A Geodynamic Approach. Special Publ. No. 76. *Geol Surv. Cairo Egypt* **1999**, *76*.

18.	Fathy, D.; Wagreich, M.; Ntaflos, T.; Sami, M. Provenance Characterization of Campanian Lacustrine Organic-Rich Mudstones on the Southern Tethyan Margin, Egypt. *ACS Earth Space Chem.* **2021**, *5*, 197–209, doi:10.1021/ACSEARTHSPACECHEM.0C00245.

19.	Fathy, D.; Wagreich, M.; Zaki, R.; Mohamed, R.S.A.; Gier, S. Geochemical Fingerprinting of Maastrichtian Oil Shales from the Central Eastern Desert, Egypt: Implications for Provenance, Tectonic Setting, and Source Area Weathering. *Geol. J.* **2018**, *53*, 2597–2612, doi:10.1002/GJ.3094.

20.	Khalil, S.; Geology, K.M.-M. and P.; 2009, Structural Control on Syn-Rift Sedimentation, Northwestern Red Sea Margin, Egypt. *Elsevier*.

21.	Fathy, D.; Wagreich, M.; Ntaflos, T.; Sami, M., Paleoclimatic Variability in the Southern Tethys, Egypt: Insights from the Mineralogy and Geochemistry of Upper Cretaceous Lacustrine Organic-Rich Deposits. Cretacous Res. **2021**, 126, 104880.

22.	Ali, S.; Ntaflos, T.; Sami, M. Geochemistry of Khor Um-Safi Ophiolitic Serpentinites, Central Eastern Desert, Egypt: Implications for Neoproterozoic Arc-Basin System in the Arabian-Nubian Shield. *Geochemistry* **2021**, *81*, 125690, doi:10.1016/j.chemer.2020.125690.

23.	El-Taher, A.; Al-Zahrani, J.H. Radioactivity Measurements and Radiation Dose Assessments in Soil of Al-Qassim Region, Saudi Arabia. *Indian J. Pure Appl. Phys.* **2014**, *52*, 147–154.

24.	Mostafa, A.M.A.; Uosif, M.A.M.; Elsaman, R.; Alrowaili, Z.A.; Moustafa, E. sayed The Dependence of Natural Radioactivity Levels and Its Radiological Hazards on the Texture of Agricultural Soil in Upper Egypt. *Environ. Earth Sci.* **2020**, *79*, 228, doi:10.1007/s12665-020-08946-z.

25.	UNSCEAR *UNSCEAR Report 2000, Sources and Effects of Ionizing Radiation, Vol. I Sources, Annex B, Exposures from Natural Radiation Sources*; United Nations, 2000.

26.	Beretka, J.; Mathew, P.J. Natural Radioactivity of Australian Building Materials, Industrial Wastes and by-Products. *Health Phys.* **1985**, *48*, 87–95, doi:10.1097/00004032-198501000-00007.

27.	Scott, D. UNSCEAR Report (1988) Sources, Effects and Risks of Ionising Radiation. United Nations Scientific Committee on the Effects of Atomic Radiation, 1988. Report to the General Assembly, with Annexes. *Int. J. Radiat. Biol.* **1989**, *55*, 1047–1048, doi:10.1080/09553008914551081.

28.	El-Taher, A.; Althoyaib, S.S. Natural Radioactivity Levels and Heavy Metals in Chemical and Organic Fertilizers Used in Kingdom of Saudi Arabia. *Appl. Radiat. Isot.* **2012**, *70*, 290–295, doi:10.1016/j.apradiso.2011.08.010.

29.	Abed, N.S.; Monsif, M.A.; Zakaly, H.M.H.; Awad, H.A.; Hessien, M.M.; Yap, C.K. Assessing the Radiological Risks Associated with High Natural Radioactivity of Microgranitic Rocks: A Case Study in a Northeastern Desert of Egypt. *Int. J. Environ. Res. Public. Health* **2022**, *19*, doi:10.3390/ijerph19010473.

30.	Tawadros, E. *Geology of North Africa*; 1st Edition, **2011**; <https://doi.org/10.1201/b11419>

# 31. Tantawy, A.; Keller, G.; Adatte, T.; [Stinnesbeckd](https://www.sciencedirect.com/science/article/pii/S0195667101902915#!), [A., Kassabe](https://www.sciencedirect.com/science/article/pii/S0195667101902915#!), [P., Schulte](https://www.sciencedirect.com/science/article/pii/S0195667101902915#!) W. Maastrichtian to Paleocene Depositional Environment of the Dakhla Formation, Western Desert, Egypt: Sedimentology, Mineralogy, and Integrated Micro-And macrofossil biostratigraphies. Cretacous Res. 2001, 22, 795-827.

32.	Hamama, H.; Kassab, A.S. Upper Cretaceous Ammonites of Duwi Formation in Gabal Abu Had and Wadi Hamama, Eastern Desert, Egypt.. J. Afric. Earth Sci. 1990, 10, 453-464.

33.	El-Ayyat, A.; Sciences, A.K.-J. of A.E.; 2004, undefined Biostratinomy and Facies Analysis of the Upper Cretaceous Oyster Storm Shell Beds of the Duwi Formation, Qusseir District, Red Sea Region, Egypt. *Elsevier*.

34.	Baioumy, H.; Research, R.T.-C.; 2005, undefined Origin of Late Cretaceous Phosphorites in Egypt. *Elsevier*.

35.	Fathy, D.; Wagreich, M.; Gier, S.; … R.M.-P.; 2018, Maastrichtian Oil Shale Deposition on the Southern Tethys Margin, Egypt: Insights into Greenhouse Climate and Paleoceanography. *Elsevier*.

36.	Baturin, C.N.; Kochenov, A.V. Uranium in Phosphorites. *Lithol Min Resour* **2001**, *36*, 303–321.

37.	Taylor, S.R.; McLennan, S.M. *The Continental Crust: Its Composition and Evolution*; 1985;

38.	Rudnick, R.L.; Gao, S. *Composition of the Continental Crust*; 2003; Vol. 3–9; ISBN 978-0-08-054807-4.

39.	Khan, K.F.; Dar, S.A.; Khan, S.A. Rare Earth Element (REE) Geochemistry of Phosphorites of the Sonrai Area of Paleoproterozoic Bijawar Basin, Uttar Pradesh, India. *J. Rare Earths* **2012**, *30*, 507–514, doi:10.1016/S1002-0721(12)60081-7.

40.	Khater, A.E.M.; Galmed, M.A.; Nasr, M.M.; El-Taher, A. Uranium and Rare Earth Elements in Hazm El-Jalamid Phosphate, Saudi Arabia: Concentrations and Geochemical Patterns Comparison. *Environ. Earth Sci.* **2016**, *75*, 1–12, doi:10.1007/s12665-016-6063-x.

41.	Abbasi, A.; Zakaly, H.M.H.; Mirekhtiary, F. Baseline Levels of Natural Radionuclides Concentration in Sediments East Coastline of North Cyprus. *Mar. Pollut. Bull.* **2020**, *161*, doi:10.1016/j.marpolbul.2020.111793.

42.	Alharshan, G.A.; Kamar, M.S.; Lasheen, E.S.R.; Ene, A.; Uosif, M.A.M.; Awad, H.A.; Issa, S.A.M.; Zakaly, H.M.H. Distribution of Radionuclides and Radiological Health Assessment in Seih-Sidri Area, Southwestern Sinai. *Int. J. Environ. Res. Public. Health* **2022**, *19*, 10717, doi:10.3390/ijerph191710717.

43.	Awad, H.A.M.; Zakaly, H.M.H.; Nastavkin, A.V.; El-Taher, A. Radiological Implication of the Granitoid Rocks and Their Associated Jasperoid Veins, El-Missikat Area, Central Eastern Desert, Egypt. *Int. J. Environ. Anal. Chem.* **2020**, 1–14, doi:10.1080/03067319.2020.1845666.

44.	Kamar, M.S.; Salem, I.A.; El-Aassy, I.E.; El-Sayed, A.A.; Zakaly, H.M.H.; Alzahrani, A.M.; Lasheen, E.S.R. An Extended Investigation of High-Level Natural Radioactivity and Geochemistry of Neoproterozoic Dokhan Volcanics: A Case Study of Wadi Gebeiy, Southwestern Sinai, Egypt. *Sustainability* **2022**, *14*, 9291, doi:10.3390/su14159291.

45.	Lasheen, E.S.R.; Azer, M.K.; Ene, A.; Abdelwahab, W.; Zakaly, H.M.H.; Awad, H.A.; Kawady, N.A. Radiological Hazards and Natural Radionuclide Distribution in Granitic Rocks of Homrit Waggat Area, Central Eastern Desert, Egypt. *Materials* **2022**, *15*, 4069, doi:10.3390/ma15124069.

## 46. Palomo, M.; Penalver, A.; Aguilar, C.; Borrull, F. Presence of Naturally Occurring Radioactive Materials in Sludge Samples from Several Spanish Water Treatment Plants. [Journal of Hazardous Materials](https://www.sciencedirect.com/journal/journal-of-hazardous-materials). **2010**. 181, 716-721.

47.	Abdelbary, H.M.; Elsofany, E.A.; Mohamed, Y.T.; Abo-Aly, M.M.; Attallah, M.F. Characterization and Radiological Impacts Assessment of Scale TENORM Waste Produced from Oil and Natural Gas Production in Egypt. *Environ. Sci. Pollut. Res.* **2019**, *26*, 30836–30846, doi:10.1007/s11356-019-06183-x.

48.	Attallah, M.F.; Hilal, M.A.; Mohamed, Y.T. Preliminary Investigations on Reducing the High Radiation Risk Level of TENORM Scale Waste from Petroleum Industry. *Radiochim. Acta* **2018**, *106*, 793–800, doi:10.1515/ract-2017-2904.

49.	Attallah, M.F.; El Afifi, E.M.; Awwad, N.S.; Aly, H.F. Comparative Study on the Radioactivity of TE-NORM in Different Components of Oil Separator Tanks. *Radiochim. Acta* **2013**, *101*, 57–65, doi:10.1524/ract.2013.1996.

50.	Attallah, M.F.; Imam, D.M. Green Approach for Radium Isotopes Removal from TENORM Waste Using Humic Substances as Environmental Friendly. *Appl. Radiat. Isot.* **2018**, *140*, 201–208, doi:10.1016/j.apradiso.2018.07.019.

51.	El-Didamony, H.; Ali, M.M.; Awwad, N.S.; Attallah, M.F.; Fawzy, M.M. Radiological Characterization and Treatment of Contaminated Phosphogypsum Waste. *Radiochemistry* **2013**, *55*, 454–459, doi:10.1134/S106636221304019X.

52.	Uosif, M.A.M.; El-Taher, A. Radiological Assessment of Abu-Tartur Phosphate, Western Desert Egypt. *Radiat. Prot. Dosimetry* **2007**, *130*, 228–235, doi:10.1093/rpd/ncm502.

53.	Makweba, M.M.; Holm, E. The Natural Radioactivity of the Rock Phosphates, Phosphatic Products and Their Environmental Implications. *Sci. Total Environ.* **1993**, *133*, 99–110, doi:10.1016/0048-9697(93)90115-M.

54.	Abbady, A.G.E.; Uosif, M.A.M.; El-Taher, A. Natural Radioactivity and Dose Assessment for Phosphate Rocks from Wadi El-Mashash and El-Mahamid Mines, Egypt. *J. Environ. Radioact.* **2005**, *84*, 65–78, doi:10.1016/j.jenvrad.2005.04.003.

55.	Hussein, E.M. Radioactivity of Phosphate Ore, Superphosphate, and Phosphogypsum in Abu-Zaabal Phosphate Plant, Egypt: *Health Phys.* **1994**, *67*, 280–282, doi:10.1097/00004032-199409000-00010.

56.	Uosif, M.A.M.; El-taher, A. Radiological Assessment of Abu-Tartur Phosphate, Western Desert Egypt. *Radiat. Prot. Dosimetry* **2008**, *130*, 228–235, doi:10.1093/rpd/ncm502.
